# Autologous platelet-rich fibrin stimulates canine periodontal regeneration

**DOI:** 10.1038/s41598-020-58732-x

**Published:** 2020-02-05

**Authors:** Chatvadee Kornsuthisopon, Nopadon Pirarat, Thanaphum Osathanon, Chanin Kalpravidh

**Affiliations:** 1 Department of Veterinary Surgery, Faculty of Veterinary Science, Chulalongkorn University, Bangkok, 10330 Thailand; 2 Wildlife Exotic and Aquatic Pathology-Research Unit, Department of Pathology, Faculty of Veterinary Science, Chulalongkorn University, Bangkok, 10330 Thailand; 3 Center of Excellence for Regenerative Dentistry and Genomics and Precision Dentistry Research Unit, Faculty of Dentistry, Chulalongkorn University, Bangkok, 10330 Thailand

**Keywords:** Reverse transcription polymerase chain reaction, Gingivitis

## Abstract

Platelet-rich fibrin (PRF) provides a scaffold for cell migration and growth factors for promoting wound healing and tissue regeneration. Here, we report using PRF in periodontal healing after open flap debridement (OFD) in canine periodontitis. A split-mouth design was performed in twenty dogs. Forty periodontitis surgical sites were randomly categorized into 2 groups; OFD alone and OFD with PRF treatment. Clinical parameters of periodontal pocket depth, gingival index, and the cemento-enamel junction-alveolar bone levels/root length ratio were improved in the OFD + PRF group. The OFD + PRF group also demonstrated a dramatically decreased inflammatory score compared with the OFD group. Collagen accumulation was improved in the OFD + PRF group at later time points compared with baseline. PRF application also significantly reduced inflammatory cytokine expression (*TNFA* and *IL1B*), and promoted the expression of collagen production-related genes (*COL1A1*, *COL3A1*, and *TIMP1*) and growth factors (*PDGFB*, *TGFB1*, and *VEGFA*). These findings suggest that PRF combined with OFD provides a new strategy to enhance the overall improvement of canine periodontitis treatment outcomes, especially in terms of inflammation and soft tissue healing. Therefore, PRF use in treating periodontitis could play an important role as a regenerative material to improve canine periodontitis treatment.

## Introduction

Periodontitis is an inflammatory disease of the periodontium comprising the gingiva, alveolar bone, periodontal ligament, and cementum^1^. The pathogenesis of periodontitis involves the interaction between host defense mechanisms and the dental biofilm. Triggered by microorganisms, periodontitis is caused by the chronic immune response, leading to inflammatory cytokine production that results in the destruction of the periodontium and subsequent manifestations of periodontitis^2,3^. In addition to its local impact on the periodontium and oral hygiene, periodontitis has a significant systemic effect. Previous reports have demonstrated that periodontitis is associated with obesity, diabetes, low birth weight, osteoarthritis, and cardiovascular disease^4,5^. In periodontitis treatment, the aim of the first phase is to eliminate the infectious source by removing plaque and calculus. Concomitantly, appropriate oral hygiene behavior is required to maintain a healthy periodontal status. In patients with periodontal destruction, periodontal regenerative treatments are commonly performed to restore the function of the periodontium^6,7^.

Various clinical modalities have been introduced as periodontal regenerative treatments. These treatments have focused on down-regulating inflammation, stimulating periodontal regeneration, and achieving optimal oral health^8^. Autogenous bone grafts are considered the best method for bony defect regeneration; however, this method has several disadvantages. Postoperative donor site morbidity, surgical complications, severe pain, and high cost are major limitations^9^. These disadvantages led to the development of novel tissue engineering therapies as autogenous graft substitutes. Among these therapies, bone substitutes, biomaterial scaffolds, and growth factors and have been evaluated in clinical studies over the past decade^10^. Several bone substitutes have been widely investigated. However, some limitations have been reported. The potential risk of cross-infection and immunological responses from the recipient are also important reasons for using natural transplants (allografts and xenografts). When using synthetic materials (alloplasts), limited periodontal regeneration has been observed^8,11^. A cellulose-based porous matrix biomaterial has been widely investigated. This material’s osteoconductivity has been demonstrated in various reports^12^. Moreover, a study revealed that pretreating this matrix with a calcium compound solution resulted in the formation of a hydroxyapatite layer, which increased the adhesion and proliferation of human osteoblast cells *in vitro*^13^. The use of recombinant human bone morphogenetic protein type 2 (rhBMP-2) has been reported to successfully achieve sinus floor, extraction socket, and alveolar ridge augmentation^14^. Combining rhBMP-2 with other conventional methods, such as a sandwich osteotomy technique, exhibited a significant increase in bone height compared with this technique alone^15^. However, a report has demonstrated that an additional biomaterial matrix, such as an absorbable collagen sponge (ACS), is required as a delivery system to attain maximal growth factor efficacy^16^. These modalities are considered to be promising approaches; however, sophisticated materials are required and high cost is a concern.

Platelets provide various growth factors that are key participants in tissue healing and regeneration. Various matrices, including fibrin, fibronectin, and vitronectin, contain adhesion domains and are involved in cell migration. Hence, platelet-related products have been developed for use in tissue repair and regeneration treatment, especially for periodontal wound healing^17^. Platelet-rich fibrin (PRF) is the second generation platelet derivative. PRF is a physiological bioscaffold rich in integrated platelets and leukocyte cytokines that are essential for regeneration and healing. When an anti-coagulant is not added, a slow and naturally polymerizing fibrin mesh develops, leading to the formation of a fibrin network that favors cytokine entrapment and cell migration^18–20^. PRF has been widely applied in human research due to its properties of being simple, autologous, and economical. The beneficial effects of PRF have been shown in intrabony defects, furcation defects, gingival recession, and extraction socket management^21,22^. The findings of a systematic review and meta-analysis suggested that PRF use may improve alveolar ridge preservation and bone fill in extraction sockets. However, the current evidence is insufficient to conclude the benefit of PRF in bone regeneration^23^. Another meta-analysis of twelve studies reveals that PRF addition to open flap debridement improves soft and hard tissue healing as determined by the amount of gingival margin change and bone fill in intrabony defects^24^. Based on these meta-analyses’ findings, additional studies are required to formulate a definite conclusion regarding the role of PRF in periodontal tissue healing.

Evidence of the pathophysiological mechanism of PRF in periodontal regeneration remains limited. Therefore, this aim of this study was to evaluate the effect of PRF as an alternative approach for periodontitis treatment in a canine model as a prerequisite to its human clinical use. Clinical, radiological, and histological parameters; and inflammatory cytokine expression were evaluated to determine the effect of PRF and the influence of this effect on the outcome of periodontitis treatment.

## Results

### PRF improved clinical periodontal parameters

**Figure 1:**
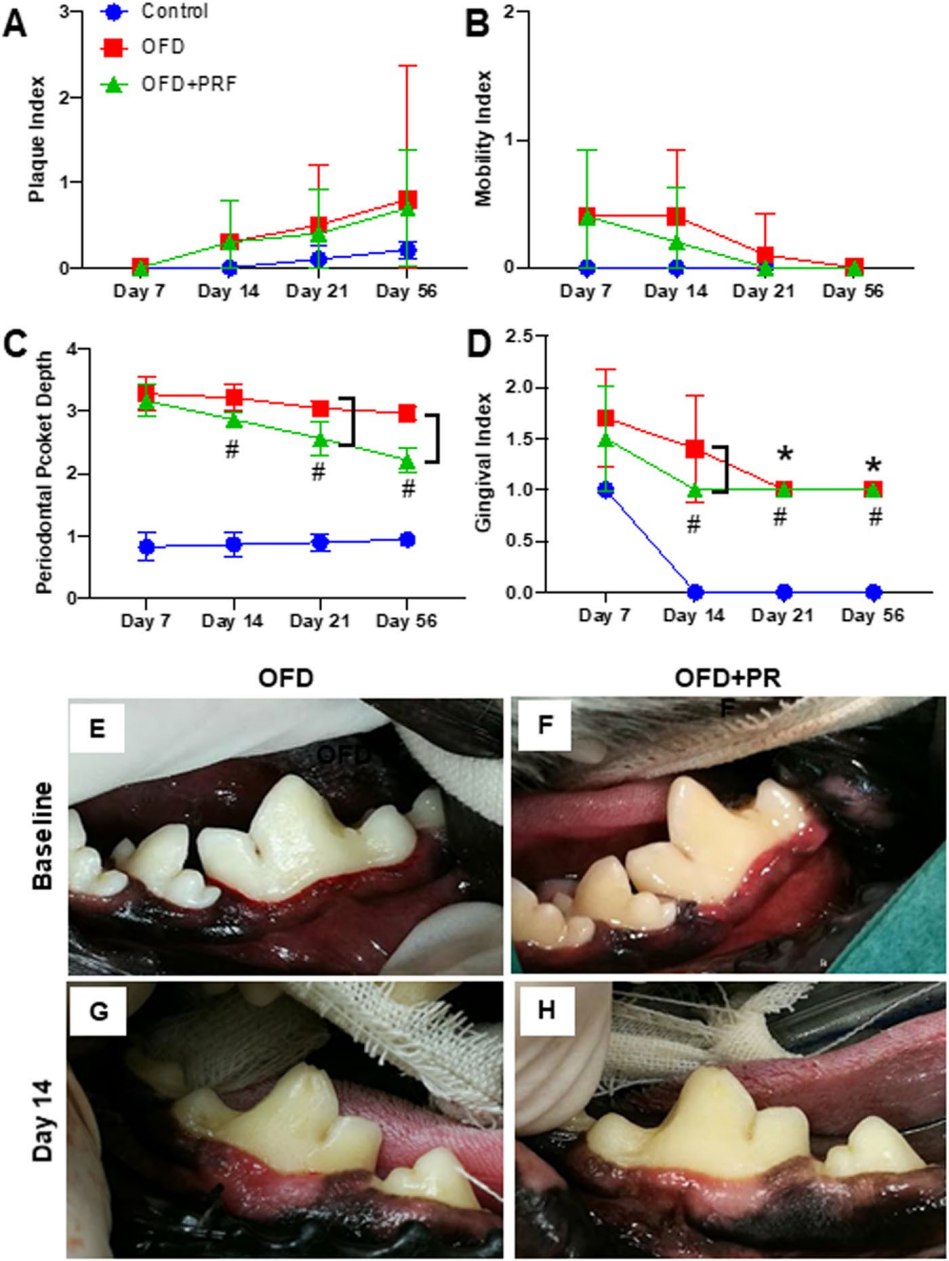
Platelet-rich fibrin (PRF) improved clinical periodontal parameters. Plaque index (**A**), mobility index (**B**), periodontal pocket depth (**C**), and gingival index (**D**) were examined on day 7, 14, 21, and 56 after surgery. Representative intraoral images of the gingival condition in the OFD and OFD+ PRF groups at baseline (**E**,**F**) and day 14 after surgery and treatment. (**G**,**H**) The control was the sham operation in dogs with healthy periodontium. OFD represents the group that exhibited periodontal disease and were treated with open flap debridement alone. OFD+ PRF refers to the group that exhibited periodontal disease and were treated with open flap debridement and PRF application. Asterisks (*) indicate significant differences between the OFD group at subsequent time points compared with the same group at day 7. Sharp (^#^) indicates significant difference between the OFD + PRF group at subsequent time points compared with the same group at day 7. Bars indicate significant differences between groups.

The OFD and OFD + PRF groups exhibited a slightly higher PI compared with the control group. However, the difference between the groups was not significant (Fig. 1A). In addition, the MI results were not significantly different between these groups at the evaluated time points (Fig. 1B). The periodontal pockets were markedly deeper in the OFD and OFD + PRF groups at all-time points compared with the control oral healthy dogs (Fig. 1C). A trend of decreased periodontal pocket depth was observed in the OFD and OFD + PRF groups. However, the periodontal pocket depth in the OFD group was not significantly different between time points. In contrast, the OFD + PRF group demonstrated a significantly decreased periodontal pocket depth at day 14, day 21, and day 56 compared with day 7. The periodontal pocket depth was significantly lower in the OFD + PRF group at day 21 and 56 compared with the OFD group. A trend of decreased GI was noted in all groups (Fig. 1D). The OFD group exhibited a significantly decreased GI at day 21 and 56 compared with day 7. In contrast, the GI in the OFD + PRF group was significantly decreased beginning at day 14. Further, a significantly decreased GI was observed in the OFD + PRF group compared with the OFD group at day 14; however, there were no significant differences between these groups at the other time points evaluated. Representative images of the clinical gingival status in the OFD and OFD + PRF groups are illustrated in Fig. 1E–H.

### PRF did not affect alveolar bone gain

**Figure 2:**
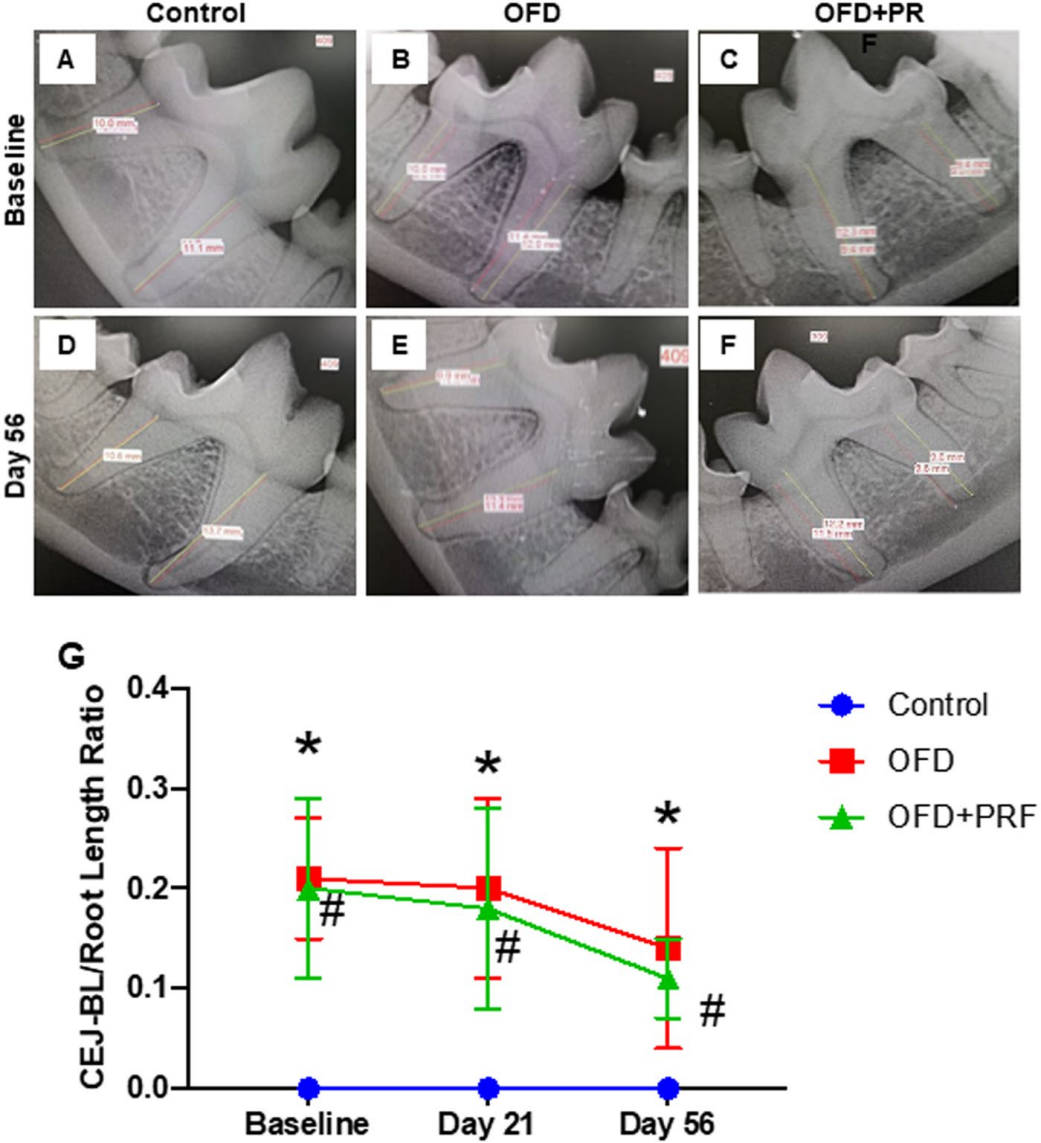
Platelet-rich fibrin (PRF) did not affect alveolar bone gain. Representative radiographic images of the alveolar bone loss in the control, OFD, and OFD + PRF groups at baseline (**A**–**C**) and day 56 after surgery and treatment. (**D**–**F**) The cemento-enamel-alveolar bone levels (CEJ-BL)/root length ratio was calculated. (**G**) The control was the sham operation in dogs with healthy periodontium. OFD represents the group that exhibited periodontal disease and were treated with open flap debridement alone. OFD + PRF refers to the group that exhibited periodontal disease and were treated with open flap debridement and PRF application. Asterisks (*) indicate significant difference between the OFD group at subsequent time points compared with the same group at the baseline. Sharp (^#^) indicate significant difference between the OFD + PRF group at subsequent time points compared with the same group at base line.

Representative radiographic images of each group are presented in Fig. 2A–F. A decreased CEJ-BL/root length ratio was observed in the OFD and OFD + PRF groups at day 21 and 56 day compared with baseline (Fig. 2G). This ratio was slightly lower in the OFD + PRF group than that in the OFD group. However, these ratios were not significantly different.

### PRF reduced the inflammatory reaction and ameliorated fibrosis

**Figure 3:**
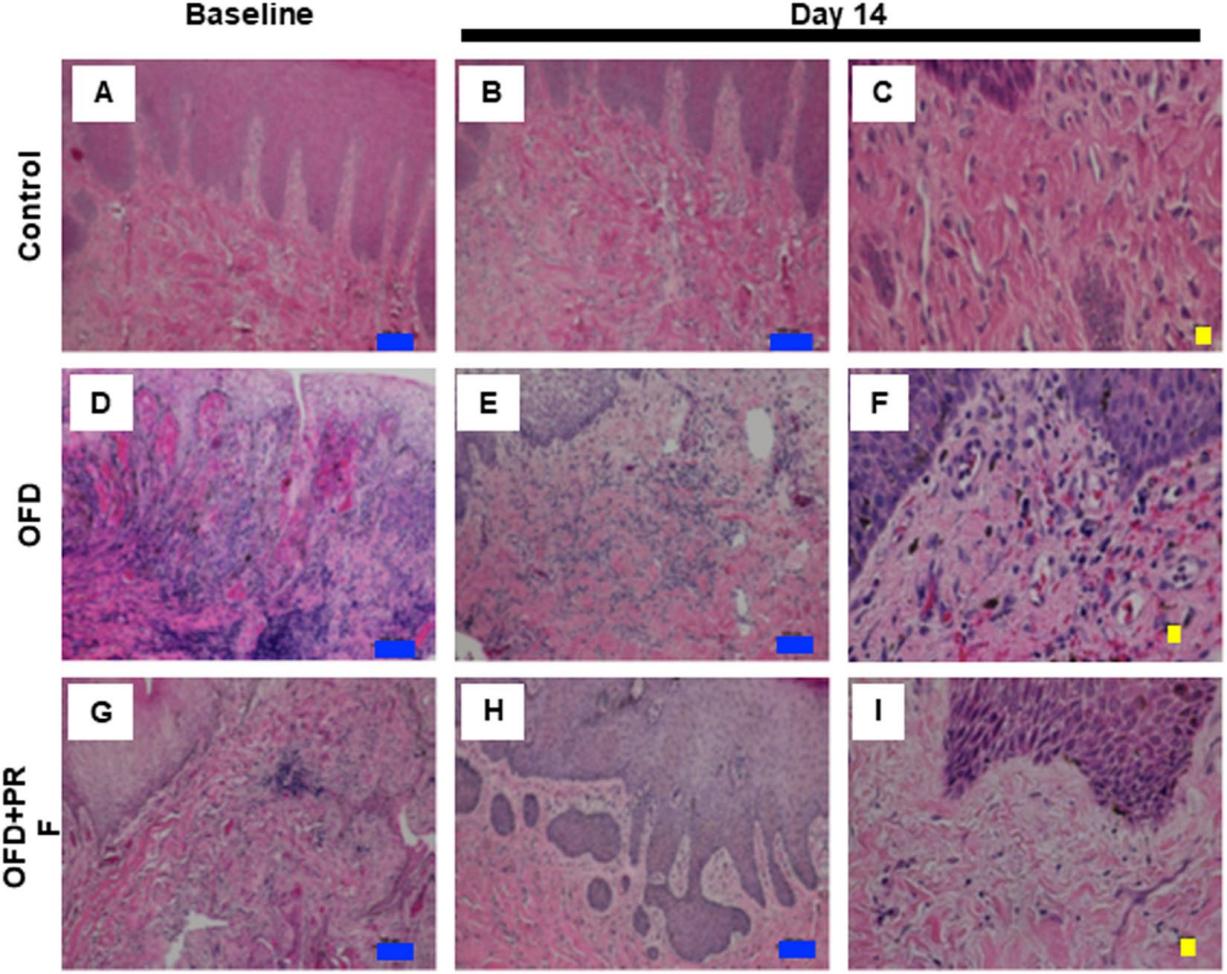
Effect of Platelet-rich fibrin (PRF) application on gingival inflammation. Gingival tissue biopsies were collected at baseline and day 14 after surgery and treatment. Tissues were processed for histological analysis and stained with hematoxylin and eosin. The control was the sham operation in dogs with healthy periodontium. OFD represents the group that exhibited periodontal disease and were treated with open flap debridement alone. OFD + PRF refers to the groups that exhibited periodontal disease and were treated with open flap debridement and PRF application. Blue and yellow bars indicate 100 μm and 10 μm, respectively.

**Figure 4:**
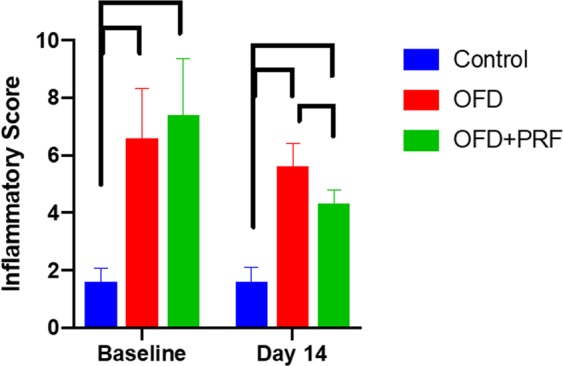
Effect of Platelet-rich fibrin (PRF) application on gingival inflammation. The inflammatory score was calculated from tissue samples at day 14 after surgery and treatment. The control was the sham operation in dogs with healthy periodontium. OFD represents the groups that exhibited periodontal disease and were treated with open flap debridement alone. OFD + PRF refers to the groups that exhibited periodontal diseases and were treated with open flap debridement and PRF application. Bars indicate a significant difference between groups.

**Figure 5:**
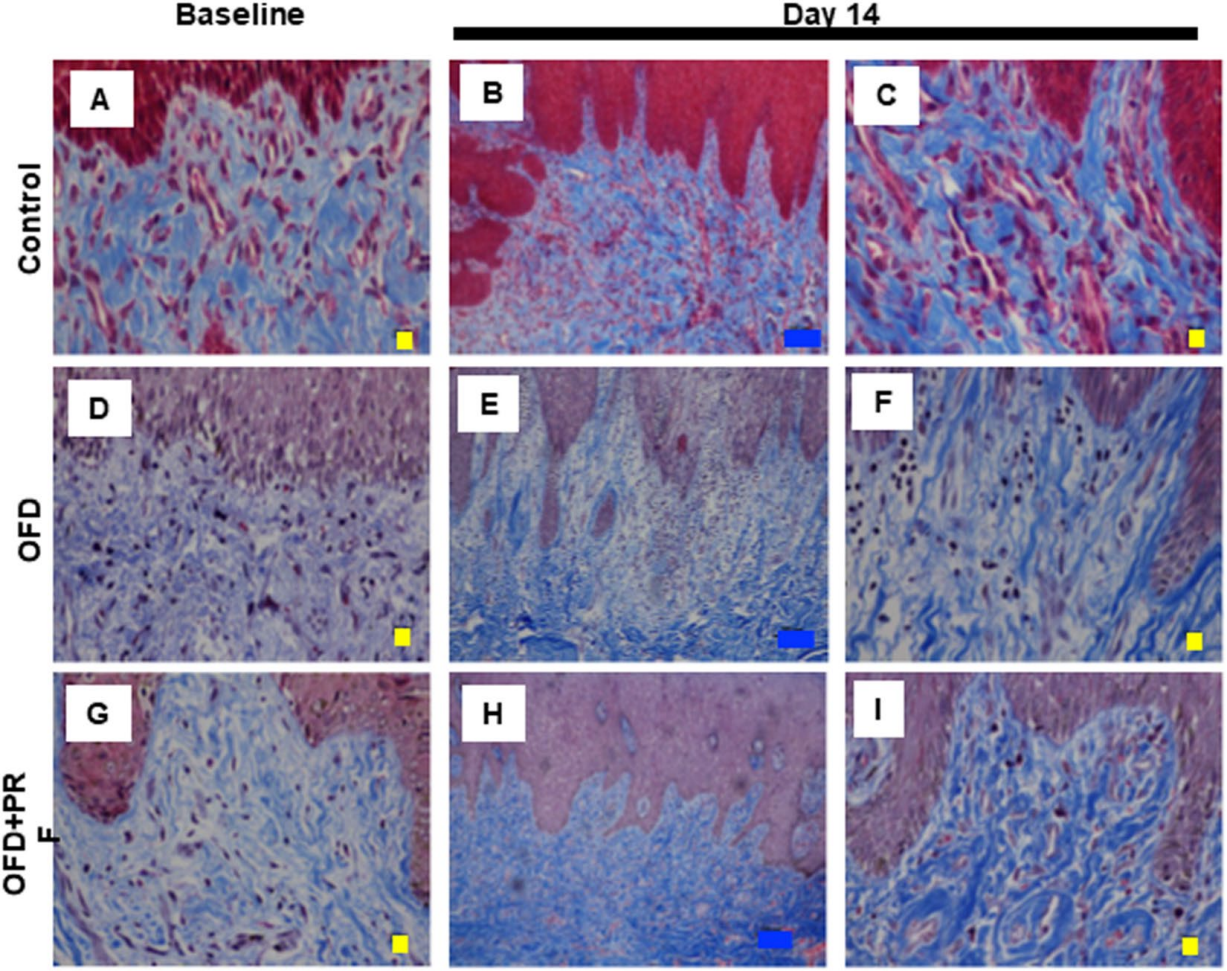
Effect of Platelet-rich fibrin (PRF) application on collagen accumulation. Gingival tissue biopsies were collected at baseline and day 14 after surgery and treatment. Tissues were processed for histological analysis and stained with Masson’s Trichrome. The control was the sham operation in dogs with healthy periodontium. OFD represents the group that exhibited periodontal disease and were treated with open flap debridement alone. OFD + PRF refers to the groups that exhibited periodontal disease and were treated with open flap debridement and PRF application. Blue and yellow bars indicate 100 μm and 10 μm, respectively.

The representative baseline histological images indicated that the OFD and OFD + PRF groups had an abundant inflammatory cell infiltration, mainly plasma cells and lymphocytes, suggesting chronic periodontitis (Fig. 3). The inflammatory score was significantly higher in the OFD and OFD + PRF groups than that of the control at baseline and day 14 (Fig. 4). At day 14, inflammatory cells were still found in the OFD + PRF group (Fig. 3H,I). However, the OFD + PRF group inflammatory score at day 14 was significantly lower compared with the OFD group (Fig. 4). Collagen accumulation was observed using Masson’s Trichrome staining. The OFD and OFD + PRF groups demonstrated loose and randomly arranged connective tissue at baseline (Fig. 5). However, at day 14, the OFD + PRF group exhibited dense and well-organized connective tissue with neovascularization.

### PRF regulated gene expression related to inflammation and healing in periodontal tissues

**Figure 6:**
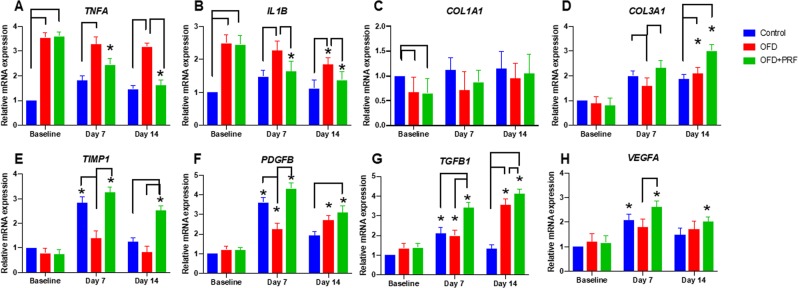
Effect of Platelet-rich fibrin (PRF) application on the expression of genes related to inflammation and periodontal healing. The mRNA expression levels were examined using real-time polymerase chain reaction. The control was the sham operation in dogs with healthy periodontium. OFD represents the group that exhibited periodontal disease and were treated with open flap debridement alone. OFD + PRF refers to the group that exhibited periodontal disease and were treated with open flap debridement and PRF application. Blue and yellow bars indicate 100 μm and 10 μm, respectively. Asterisks (*) indicate a significant difference compared with the same group at baseline.

Two major pro-inflammatory cytokines, *TNFA* and *IL1B*, were chosen to evaluate the effect of PRF treatment on inflammation. The OFD + PRF group exhibited a significant decrease in *TNFA* and *IL1B* expression at day 7 and 14 compared with baseline (Fig. 6A,B). At day 14, the *TNFA* and *IL1B* mRNA levels were significantly lower in the OFD + PRF group than those in the OFD group.

*COL1A1* mRNA expression was significantly lower in the OFD and OFD + PRF groups compared with the control at baseline (Fig. 6C). At day 7 and 14, the *COL1A1* levels were slightly lower in the OFD and OFD + PRF groups than that of control; however, the difference was not significant. The OFD group exhibited lower *COL3A1* and *TIMP1* mRNA levels compared with the control and OFD + PRF groups at day 7 (Fig. 6D,E). In contrast, a significant increase in *COL3A1* expression was observed at day 14 in the OFD and OFD + PRF groups compared with the control group (Fig. 6D). The OFD + PRF group demonstrated markedly increased *TIMP1* expression at day 14 compared with the control and OFD groups (Fig. 6E). A similar expression pattern was observed for *PDGFB* mRNA expression (Fig. 6F). Further, the OFD + PRF group exhibited significantly higher *TGFB1* mRNA expression than that of the control and OFD groups at day 7 and 14 (Fig. 6G). Moreover, *TGFB1* mRNA levels were markedly increased in the OFD and OFD + PRF groups at day 7 and 14 compared with baseline. Lastly, the *VEGFA* mRNA expression was evaluated (Fig. 6H). The OFD + PRF group demonstrated a significant increase in *VEGFA* levels at day 7 and 14 compared with baseline. In addition, a marked upregulation in *VEGFA* mRNA levels in the OFD + PRF group was observed compared with the OFD group at day 7.

### PRF contained TGF-β1 and VEGF-A

**Figure 7:**
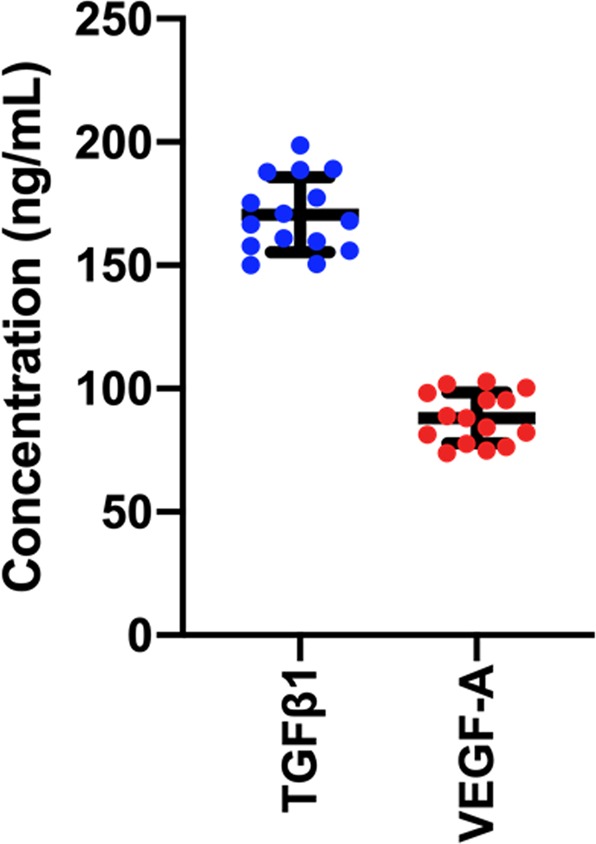
Platelet-rich fibrin (PRF) contained growth factors. The concentration of TGF-β1 and VEGF-A was evaluated using enzyme linked immunosorbent assay.

The protein expression of TGF-β1 and VEGF-A in PRF was examined using ELISA. As shown in Fig. 7, TGF-β1 protein concentration (170.50 ± 15.24 mg/ml) in PRF was higher than the VEGF-A concentration (88.08 ± 10.32 mg/ml).

## Discussion

The present study investigated the effect of PRF membranes in treating periodontitis as evaluated by various clinical, radiological, and histological parameters; and gene expression. We divided the experimental animals into 3 groups; sham operation in dogs with a healthy periodontium, OFD in dogs with periodontitis, and OFD + PRF treatment in dogs with periodontitis. In the control group, an elevated GI score was observed at day 7 due to the normal tissue response to the OFD procedure. The GI score in the control group returned to 0 at day 14, demonstrating that healthy gingiva heals rapidly. PRF treatment resulted in a decreased periodontal pocket depth and GI score compared with dogs treated with OFD alone. The present results corresponded with human studies that demonstrated that the PRF-treated group had a significantly reduced GI score and periodontal pocket depth compared with OFD only in intrabony defects^25–28^. Furthermore, the combination of an anorganic bovine bone mineral (ABBM) and PRF resulted in a reduced GI score and periodontal pocket depth compared with those treated with ABBM alone^29^. The present study also found that the PI between the OFD and OFD + PRF groups was not significantly different. Similarly, a previous report illustrated that PRF treatment with ABBM did not significantly alter the PI^29^. The explanation for this observation is that the PRF was placed over the alveolar crest, thus, the crown was not covered by the PRF. We hypothesize that this placement resulted in the non-significant difference in the PI between experimental groups. We also evaluated the MI in each group. There was no significant difference in the MI between the OFD and OFD + PRF groups in our study. This finding might be because the present study was short-term compared with human studies. It has been reported that PRF treatment resulted in decreased tooth mobility at 12- and 18-months post-treatment^30,31^.

PRF application resulted in a significant increase in radiographic density and bone fill in the PRF-treated group evaluated at 12 months post-operatively in humans^32^. Moreover, significantly higher new bone area percentages were observed in rat periodontal fenestration defects treated with PRF mixed with periodontal ligament stem cells at 2 months post-operatively as determined by histological analysis^33^. In contrast, the present study demonstrated that the alveolar bone gain was not significantly different when the PRF was applied after OFD. A previous study demonstrated that PRF treatment improved probing pocket depth, relative attachment level, and radiographic bone fill comparable to those treated with autologous bone grafts^34^. However, it was noted that the autologous bone grafts significantly promoted bone fill compared with PRF^34^. Correspondingly, a meta-analysis of PRF application as an adjuvant to open flap debridement demonstrates that PRF application increases the bone fill in intrabony defects^24^. However, the supportive evidence is not definitively conclusive^24^. Hence, additional clinical trials are required to conclude the effect of PRF on bone regeneration.

Various approaches for periodontal regeneration have been studied in several clinical investigations. Many studies observed positive results in hard tissue regeneration when using growth factors. A combination of rhBMP-2 and ACS was applied at the osteonecrotic maxillary/mandibular lesions induced by a bisphosphonate and in jaw reconstruction after tumor resection in human clinical cases. Radiographic evaluation demonstrated new bone formation by 3 or 4 months postoperatively^35,36^. Similar results were reported in a non-human primate animal model of distraction osteogenesis. This combination induced new bone formation at 3 months post-operation based on histological examination^37^. Another promising approach is using a titanium mesh. A report indicated that titanium mesh filled with autogenous bone and deproteinized anorganic bovine bone utilized as a barrier membrane enhanced alveolar ridge reconstruction^38^. Compared with our results, other approaches seem more effective compared with PRF alveolar bone regeneration. However, well-designed clinical trials and meta-analysis are required to confirm these findings.

To investigate the effect of PRF histologically, the inflammatory reaction and fibrosis score were assessed at baseline and day 14. We found that the OFD + PRF group had a significant reduction in the inflammatory reaction score. There are few studies concerning the effect of PRF on inflammation. Moreover, these studies focused on clinical and radiographic parameters to determine the efficacy of PRF in periodontal treatment, despite that histological assessment is one of the most accurate evaluation methods^26^. We hypothesized that the reduced inflammatory reaction score in the OFD + PRF group resulted from the various anti-inflammatory cytokines that are embedded in the PRF fibrin meshwork. Our results indicated that the collagen accumulation was not significantly different between the OFD and OFD + PRF groups. This observation might be attributed to the inadequate sensitivity of our histological evaluation. Other methods such as immunohistochemistry, wound healing assay, or fibroblast and collagen gene detection might generate different results.

A previous study reported that PRF prepared from beagle dogs contained TGF-β1 at a concentration of approximately 64 ng/ml^39^. However, our study demonstrated a higher TGF-β1 concentration in PRF. This difference might be due to the different preparation procedures and variation in experimental animals. Further, it has been shown that plasma and platelet-rich plasma contained lower concentrations of TGF-β1 and VEGF-A than the present study^40,41^. Therefore, this could imply that PRF contains a higher growth factor concentration compared with plasma and platelet-rich plasma due to its effective growth factor entrapment ability.

To understand the mechanism of PRF in periodontal healing, we evaluated the expression of various genes associated with inflammation and periodontal wound healing. Our results revealed that the OFD + PRF group had a significant upregulation in *TGFB1*, *PDGFB*, *VEGFA*, and *COL3A1* expression, while the *TNFA* and *IL1B* mRNA levels were downregulated. TGF-β1, PDGF-B, and VEGF are key growth factors found in PRF. TGF-β1 significantly participates in wound healing processes, including immune cell modulation, stimulating osteoblast proliferation, and promoting collagen synthesis^42^. PDGF-B is a member of the PDGF family and is a powerful chemoattractant, angiogenesis mediator, and potent activator of mesenchymal lineage cell migration and proliferation. PDGF-B mainly functions in the inflammatory and proliferative phases of wound healing^43^. VEGF acts as an endothelial mitogen, chemotactic agent, is angiogenic, and induces epithelialization and collagen deposition. VEGF predominately functions in the inflammatory and proliferative process of wound healing^44^. Therefore, high expression of these cytokines would increase the entire wound healing process. PRF has been shown to increase angiogenesis in guided-bone regeneration of cranial defects in rabbits^45^. The VEGF expression observed via immunostaining was higher in the group receiving xenogenic bone combined with PRF compared with the xenogenic bone alone group^45^.

TIMP-1, COL1A1, and COL3A1 mainly participate in the proliferative and remodeling phases of periodontal wound healing. TIMP functions as a matrix metalloproteinase (MMP) inhibitor, and plays an important role in the remodeling phase by inhibiting extracellular matrix and collagen breakdown^46^. Collagen is a basic component of the periodontium. Type III collagen is predominately synthesized during the initial phase of wound healing and is then gradually replaced with type I collagen 2‒3 weeks after the initiation of wound healing^47^. We found that *TIMP1* and *COL3A1* mRNA expression was significantly higher in the OFD + PRF group. Similarly, it has been shown that PRF combined with periodontal ligament stem cells and jaw bone mesenchymal stem cell sheets exhibited higher *COL1A1* and *COL3A1* expression compared with control^48^. Based on their function and expression in our study, we concluded that their high expression in the OFD + PRF group in conjunction with the growth factors mentioned above accelerated periodontal healing. The expression of *COL1A1* and *COL3A1* mRNA in the OFD + PRF group correlates with our histological observation of dense collagen fiber accumulation at day 14.

TNF-α and IL-1β are key pro-inflammatory cytokines that are involved in the pathogenesis of periodontitis and decrease wound healing and regeneration^49^. High production of these cytokines stimulates other inflammatory mediators, tissue destruction by MMP induction, and bone resorption by stimulating osteoclast activity^50,51^. Therefore, high expression would result in increased inflammation and tissue destruction. Our results revealed that the OFD group presented higher expression of these cytokines, which was related to the higher GI and inflammatory reaction score compared with OFD + PRF group. Deeper periodontal pockets might also be associated with our cytokine expression results.

Based on our overall results, PRF potentiates wound healing and diminishes the inflammatory response. The upregulation of *TGFB1*, *PDGFB*, *VEGFA*, *TIMP1*, *COL1A1*, and *COL3A1* could act as chemo-attractants for other immune cells and fibroblasts, inhibiting extracellular matrix degradation, promoting angiogenesis, inducing cell proliferation, and stimulating collagen and extracellular matrix synthesis. Likewise, stimulating inflammatory mediator production, MMP expression, matrix producing cell apoptosis, and osteoclast activity might also be diminished due to the downregulated *TNFA* and *IL1B* expression. These PRF effects can explain the increased periodontal attachment gain, decreased gingivitis, and decreased histological inflammatory score found in our study.

The PRF preparation procedure used in our study differed from that of most studies. First, the present study collected 4 mL of autologous venous blood. Thus, the PRF characteristics in the present study may differ from other studies using 10 mL of blood. The 4 mL volume was chosen due to the limited periodontal defect size in the canine periodontitis model. Our preliminary study demonstrated that the application of PRF collected from 10 mL autologous blood volume in the defect resulted in gingival flap dehiscence at day 1 or 3 post-operatively. This gingival flap dehiscence could affect the healing processes and compromise the interpretation of our results. In addition, it has been shown that platelet and leukocyte distribution in the PRF membrane is different at specific regions^52,53^. Therefore, PRF prepared from 10 mL blood and cutting a relatively large portion out to fit in the canine defect may not contain the same components as did our PRF, causing less effects, in the present study. Further, PRF preparation from 5 mL blood has previously been reported and utilized in both human and dogs^54–56^. Therefore, we decided to use 4 ml of blood, which resulted in a relatively appropriate PRF size that fit the defect size and gingival flap in our canine model. Second, the centrifugation method was modified from the conventional method. In the present study, reduced relative centrifugal forces (RCF) and time was used. Reducing the RCF results in increased cell populations in the PRF collected from the top one-third layer, whereas the high centrifugation forces used in the previous platelet-rich fibrin preparation protocol shift the cell populations to the bottom of the tubes. In addition, a lower centrifugation time reduces cell pull-down by centrifugation forces, which increases the cell populations in the platelet-rich fibrin matrix^57^. The decreased RCF and time resulted in significant increases in platelet cell numbers, monocyte/macrophage behavior, and growth factor release compared with other preparation methods^41,57,58^. Moreover, in terms of tissue regeneration, human gingival fibroblasts demonstrated significantly increased migration and proliferation when cultured with PFR acquired using decreased centrifugation speed and time^57^. Other studies additionally revealed that reduced RCF contributed to increased leukocytes and platelets gain in PRF matrices^59–61^. Third, the centrifugation machine and the blood collection tubes are different between ours and other studies. As reported previously, different apparatuses utilized in PRF preparation may influence the PRF quality^62^. These differences would affect the RCF at the PRF clot (referred to as RCF-clot), the RCF at the shortest distance from the rotor (referred to as PRF-min), and the RCF at the largest distance from the rotor (referred to as PRF-max)^62^. As stated in the materials and methods section, 1,300 rpm for 8 min (RCF-clot = 164 g) was used in our study. This RCF-clot value was slightly different from those in another study; however, it was similar to the low-speed concept PRF that is produced using a low centrifugation speed (approximately 200 g RCF-max and 130 g RCF-clot)^57,58,63^. Another consideration is that the tube utilized for PRF preparation is crucial^58^. In the present study, a glass tube was used. Generally, platelets can interact with a glass surface, resulting in coagulation activation during centrifugation. This interaction leads to the formation of a solid PRF matrix composed of a fibrin network entrapping platelets, leukocytes, plasma proteins, and growth factors^64^. Moreover, a PRF clot made from a glass tube forms and retracts from the tube wall faster compared with a plastic tube^65^. Due to the differences between our and other studies, we measured the concentration of two key cytokines, TGF-β1 and VEGF-A to ensure that an adequate amount of these growth factors was present in each PRF membrane. We found that the mean concentration of TGF-β1 was slightly higher compared with a previous report^39^. Therefore, we assume that our PRF contained an adequate amount of growth factors.

The present study was conducted in a canine periodontitis model, which has some limitations. Although canine periodontal anatomy and its physiological mechanisms are well described, this limitation is of concern. Hence, research methodology and a periodontal defect model in dogs should be further developed to use as a predictable translatable animal model prior to human clinical trials^66^. To confirm the observations in our study, long-term and large-scale studies should be performed. Moreover, other cytokines should be investigated, especially those involving in the initiation and progression of periodontal diseases. This would help to elucidate the potential mechanism of PRF in periodontal applications. However, within the limitation of the present study, we found that PRF improves clinical outcomes, accelerates wound healing, and reduces the inflammatory response. PRF could be a novel alternative modality for periodontitis management in human and dogs. However, additional randomized control trials are required to ensure the positive effect of PRF in clinical application.

## Materials and Methods

### Experimental animals

Experimental animals were obtained from the Small Animal Teaching Hospital at Chulalongkorn University with the following inclusion criteria: 1) Mesocephalic dogs aged between 8 months to 5 years, 2) Healthy dogs based on physical examinations and laboratory tests, 3) Maxillary 4th premolars and mandibular 1st molars with periodontal pocket depth (PPD) between 3‒5 mm, and 4) No periodontal complications, e.g. fractured teeth^67^. Dogs with a history of receiving anti-inflammatory medicine within 30 d, having systemic/metabolic/immunosuppressive illness, insufficient platelet count (<20,000/mm^3^), teeth with alveolar bone loss over 75%, or a mobility index grade 2 or higher were excluded from the study^68^. Dogs with healthy oral status served as the controls (Control group, n = 5). Forty periodontitis sites were identified based on the inclusion criteria and divided using a split mouth clinical design into 2 groups; open-flap debridement (OFD group, n = 20) and OFD with PRF treatment (OFD + PRF group, n = 20). Informed consent was obtained from the owners and the experiments were conducted in accordance with the guidelines for animal welfare of experimental animals and approved by the Chulalongkorn University Animal Care and Use Committee, Pathumwan, Bangkok, Thailand (#1831017)

### Surgical procedure

**Figure 8:**
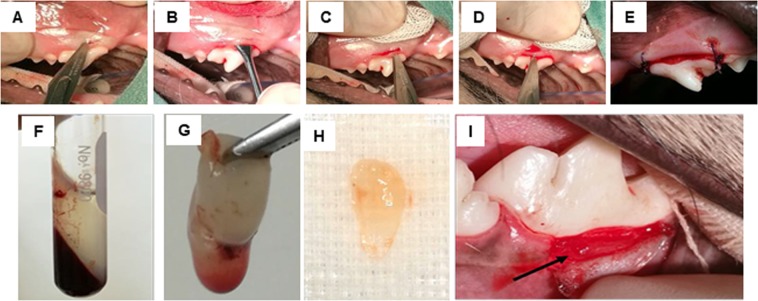
Surgical procedure and platelet-rich fibrin (PRF) preparation. The Modified Widman flap technique is shown in (**A**–**E**). An internal bevel incision was created (**A**), followed by mucoperiosteal flap reflection. (**B**) Subsequently, an intrasulcular incision (**C**) and a horizontal incision (**D**) were performed to remove the pocket epithelium. Scaling and root planning was done. Lastly, an interdental suture was used for flap closure. (**E**) For PRF preparation, blood was drawn from jugular vein and centrifuged at 1,300 rpm for 8 min. (**F**) Subsequently, the PRF was separated from the packed red blood cell layer. (**G**) The liquid in the PRF construct was removed by gentle gauze compression. (**H**) In the open flap debridement and PRF treatment (OFD + PRF) group, the PRF was placed over the alveolar bone at the cemento-enamel junction level. Black arrow indicates the PRF.

Each surgical procedure was performed under general anesthesia using intramuscular injection with 0.02 mg/kg acepromazine (2 mg/ml, Vetranquil; CEVA Sante Animal, France) and 0.3 mg/kg morphine (10 mg/ml) as a premedication. General anesthesia was induced with 2–4 mg/kg propofol (10 mg/ml, Lipuro 2%; Braun, Germany) and inhalation with 2% isofurane was used for anesthetic maintenance. Local anesthesia of the maxilla and mandible was obtained with 0.5% bupivacaine. Cefazolin (22 mg/kg) was given as a prophylaxic antibiotic^69^. Full mouth dental scaling and polishing were performed. The studied sites underwent OFD via the oft-modified Widman flap technique (MWF) (Fig. 8A–E). The procedure comprised an internal bevel incision, mucoperiosteal flap reflection, intrasulcular incision, and horizontal incision along the alveolar crest. Root planning was performed using ultrasonic instruments and Gracey curettes (Hu-Friedy Mfg Co. Inc., Chicago) without osteotomy or contouring osteoplasty. The mucoperiosteal flap was repositioned with 4–0 monofilament absorbable suture material (Monosyn®, B. Braun, Spain) using an interrupted interdental suture pattern. The suture was removed after a healing period of 7 d^70^. For post-operative care, each dog received 15 mg/kg amoxy-clavulanic acid and 4 mg/kg tramadol hydrochloride twice a day for 5 d. Chlorhexidine gluconate (0.12% v/v) was used as a mouthwash and they were fed Hill’s® Prescription Diet® a/d® Canine/Feline for 7 d.

### Platelet-rich fibrin preparation and administration

The PRF membranes were prepared based on a previous protocol with slight modification^57^. Four mL of autologous blood was collected from the jugular vein and kept in a sterile 10 ml glass tube (16 mmx100 mm, Becton, Dickinson and Company, Franklin Lakes, NJ, USA). PRF membranes were produced using 1,300 rpm for 8 min (RCF-clot = 164 g, RCF-max = 303 g) using a Kubota4000 centrifugation machine (Japan) at a 45° rotor angulation with a radius of 87 mm at the clot and 160 mm at the max. The PRF clot formed between the acellular plasma (upper layer) and red blood cell base (bottom layer) was harvested 1 mm below the interface with the red blood cell layer to maximize platelet quantity^52,71^ (Fig. 8F,G). The PRF membrane was compressed in a gauze and then cut into 4 × 5 mm pieces with a scalpel blade, and placed in the surgical sites (Fig. 8H). The serum exudate collected from the compression was used for graft material hydration, surgical site rinse, and autologous graft storage. In the OFD + PRF group, the PRF membrane was positioned over the denuded root surface just below the cemento-enamel junction (CEJ) and mucoperiosteal flap closure was performed in the same manner as for the OFD group^25,72^ (Fig. 2D). In the control group, a sham operation was performed in dogs with a healthy periodontium.

### Clinical evaluation

The plaque index (PI), gingival index (GI), mobility index (MI), and periodontal pocket depth were determined as described by Löe^73^ and Laster, *et al*.^74^. For determining the PI, iC plaque® (iM3, Australia) was used to stain the accumulated plaque as a pink layer on the tooth surface. The GI was evaluated based on the presence of gingival inflammation on the mesial, distal, buccal, and lingual surfaces. The periodontal pocket depth was recorded as the mean measurement of 6 areas around each surgical site (mesio-buccal, mid-buccal, disto-buccal, mesio-lingual, mid-lingual, and disto-lingual) using a William’s probe. The MI was based on tooth mobility using an explorer. The criteria for each parameter are shown in Table 1.

**Table 1:** Clinical parameter criteria.

***Plaque index***	
Score 0	No plaque
Score 1	Up to 25% plaque accumulation on the tooth surface
Score 2	25–50% plaque accumulation on the tooth surface
Score 3	>50% plaque accumulation on the tooth surface
***Gingival index***	
Score 0	No inflammation and healthy periodontium
Score 1	Mild inflammation, slight change in color, slight edema, and no bleeding on probing
Score 2	Moderate inflammation, moderate change in color and consistency, and bleeding on probing
Score 3	Severe inflammation, marked redness, hypertrophy, ulceration, and spontaneous bleeding
***Mobility index***	
Score 0	Normal physiology <0.2 mm
Score 1	Slightly mobile (bucco-lingual direction)
Score 2	Moderate mobility (bucco-lingual and mesio-distal direction)
Score 3	Severe mobility (bucclingual, mesio-distal and vertical direction)

### Intra-oral radiographic evaluation

Intra-oral radiographs were taken with a CR7 Vet Dental X-ray unit (iM3, Australia). Alveolar bone loss was assessed by direct measurements of the distance between the CEJ and the alveolar bone level (BL). The distance was measured at three points; the mesial, middle, and distal aspects of each tooth. The root length was also measured for calculating the CEJ-BL/root length ratio^75,76^.

### Histological analysis

Tissue samples measuring approximately 3 × 5 mm were collected from the middle buccal area via the MWF technique to orient and contain the pocket epithelium, oral epithelium, and connective tissue in the same section^77^. The samples were fixed with 10% neutral buffered formalin, processed through a graded series of ethanol, and embedded in paraffin. The sections were obtained at a 5 μm thickness and stained with hematoxylin-eosin (H&E) and Masson’s trichrome. The inflammatory reaction and fibrosis were evaluated. For the inflammation scoring, the inflammatory cells (neutrophils, lymphocytes, plasma cells, and eosinophils) were identified and individually counted as follows: Not present (0), Mild (1), Moderate (2), and Severe (3). The sum of the total scores of each classification were divided into three grades of inflammatory reaction; mild inflammation (score 0‒3), moderate inflammation (score 4‒6), and sever inflammation (score ≥ 7)^78^.

### Enzyme-linked immunosorbent assay

The PRF membranes were cut into small pieces with a scalpel blade and homogenized using disposable homogenizers (BioMasher II, Nippi, Inc., Tokyo, Japan). After centrifuging at 3000 rpm for 10 min at 4 °C, the supernatant was collected and the concentration of TGF-β1 and VEGF-A was measured using ELISA kits per the manufacturer’s protocol (Quantikine, R&D Systems, Minneapolis, MN, USA): TGF-β1 (Cat. No. MB100B) and VEGF (Cat. No. CAVE00). The concentration was calculated using a standard curve of known concentrations of the respective proteins^79^.

### Polymerase chain reaction

Gingival tissue samples were harvested from the mid-buccal area. Total cellular RNA was extracted using TRIzol reagent (Invitrogen, Carlsbad, CA, USA) and treated with RNase-Free DNase I (Qiagen, Netherland) to remove any genomic DNA. The RNA integrity and amount were evaluated using a Nanodrop spectrophotometer (Thermo Scientific, USA). Complementary DNA transcription was performed from one microgram of total RNA using a reverse transcriptase ImPromII kit (Promega, Madison, WI, USA). Real-time polymerase chain reaction was performed using a FastStart Essential DNA Green Master kit (Roche Diagnostic, USA) on a MiniOpticon real-time PCR system (Bio-Rad, USA). The data were analyzed using the 2^−ΔΔCt^ method. Target gene expression value was normalized to *ACTB* expression values and then normalized to the expression in the periodontal healthy dog control group. The primer sequences used are shown in Table 2.

**Table 2:** Oligonucleotide sequences.

Gene	Oligonucleotide sequences	Annealing temperature (°C)	Amplified product size (bp)	Accession no.
*TGFB1*	F: 5′-GGACTTCGAGCAGGAGATGG-3′ R: 5′-TTCCATGCCCAGGAAGGAAG-3′	57	136	NM_001003309.1
*VEGFA*	F: 5′-CCGGTATAAACCCTGGAGCG-3′ R: 5′-GCAACGCGAGTCTGTGTTTT-3′	55	115	NM_001003175.2
*PDGFB*	F: 5′-ACCGGAAGTTCAAGCACACA-3′ R: 5′-TGCCCTCAATCTCCTCCAGA-3′	55	84	NM_001003383.1
*COL1A1*	F: 5′-GGCAGGAGGGTTCAGCTAAG-3′ R: 5′-GCAACAAAGTCCGCGTATCC-3′	57	160	AF153062.1
*COL3A1*	F: 5′-TTCCTGGGAGAAATGGCGAC-3′ R: 5′-AGGACCAGTAGGGCAGGATT-3′	59	98	HM775210.1
*TIMP1*	F: 5′-GATGTTCAAGGGTTTCAGCG-3′ R: 5′-TGTCACTCTGCAGTTGCAG-3′	55	294	AF077817_1
*TNFA*	F: 5′-TCTCGAACCCCAAGTGACAAG-3′ R: 5′-CAACCCATCTGACGGCACTA-3′	59	152	NM_001003244
*IL1B*	F: 5′-CAAGTCTCCCACCAGCTCTGTA-3′ R: 5′-GGGCTTCTTCAGCTTCTCCAA-3′	59	80	NM_001037971
*ACTB*	F: 5′-AGCTCCACGGAGAAGAACTG-3′ R: 5′-GGCTCCAAATGTAGGGGCAG-3′	57	148	NM_001195845.2

### Statistical analyses

The clinical, intra-oral radiographic, histological, and cytokine expression data are expressed as mean ± S.D. The intergroup and intragroup comparisons of specific parameters were evaluated using two-way ANOVA followed by the Bonferroni-type multiple *t*-test. The data were quantitatively analyzed using SPSS version 22 for Windows program (Version 22, IBM, US). A significant difference was considered when *P* < 0.05.

## References

[1] Albuquerque C (2012). Canine periodontitis: the dog as an important model for periodontal studies. Vet. J..

[2] Kriebel K, Hieke C, Müller-Hilke B, Nakata M, Kreikemeyer B (2018). Oral Biofilms from Symbiotic to Pathogenic Interactions and Associated Disease -Connection of Periodontitis and Rheumatic Arthritis by Peptidylarginine Deiminase. Front. Microbiol..

[3] How KY, Song KP, Chan KG (2016). Porphyromonas gingivalis: An Overview of Periodontopathic Pathogen below the Gum Line. Front. Microbiol..

[4] Kuo LC, Polson AM, Kang T (2008). Associations between periodontal diseases and systemic diseases: a review of the inter-relationships and interactions with diabetes, respiratory diseases, cardiovascular diseases and osteoporosis. Public. Health.

[5] Yamashita JM (2015). Assessment of Oral Conditions and Quality of Life in Morbid Obese and Normal Weight Individuals: A Cross-Sectional Study. PLoS One.

[6] Matthews DC (2014). Prevention and Treatment of Periodontal Diseases in Primary Care. Evid. Based Dent..

[7] Mombelli A (2018). Microbial colonization of the periodontal pocket and its significance for periodontal therapy. Periodontology 2000.

[8] Carmagnola D, Tarce M, Dellavia C, Rimondini L, Varoni EM (2017). Engineered Scaffolds and Cell-Based Therapy for Periodontal Regeneration. J. Appl. Biomater. Funct. Mater..

[9] Wang W, Yeung KWK (2017). Bone grafts and biomaterials substitutes for bone defect repair: A review. Bioact. Mater..

[10] Fujioka-Kobayashi M (2017). Absorbable collagen sponges loaded with recombinant bone morphogenetic protein 9 induces greater osteoblast differentiation when compared to bone morphogenetic protein 2. Clin. Exp. Dent. Res..

[11] Sheikh Z (2017). Natural graft tissues and synthetic biomaterials for periodontal and alveolar bone reconstructive applications: a review. Biomater. Res..

[12] Hickey RJ, Pelling AE (2019). Cellulose Biomaterials for Tissue Engineering. Front. Bioeng. Biotechnol..

[13] Petrauskaite O (2013). Biomimetic Mineralization on a Macroporous Cellulose-Based Matrix for Bone. Regeneration. BioMed. Res. Int..

[14] Cicciù, M., Scott, A., Cicciù, D., Tandon, R. & Maiorana, C. Recombinant Human Bone Morphogenetic Protein-2 Promote and Stabilize Hard and Soft Tissue Healing for Large Mandibular New Bone Reconstruction Defects. *Journal of Craniofacial Surgery* **25** (2014). 10.1097/SCS.0000000000000830 24820713

[15] Herford, A. S., Tandon, R., Stevens, T. W., Stoffella, E. & Cicciu, M. Immediate Distraction Osteogenesis: The Sandwich Technique in Combination With rhBMP-2 for Anterior Maxillary and Mandibular Defects. *Journal of Craniofacial Surgery* **24** (2013). 10.1097/SCS.0b013e318292c2ce 23851812

[16] Sudharsana A, Arjunkumar R (2015). Recombinant human bone morphogenetic protein 2/Absorbable collagen sponge (rhBMP-2/ACS) in periodontal therapy- An Overview. J. Pharm. Sci. Res..

[17] Srivastava R, Mukherjee S, Saxena S, Saha S (2019). A Novel Healing Platelet Rich Fibrin (PRF) Matrix and its Role in Dentistry. J. Adv. Med. Dental Sci. Res..

[18] Dohan DM (2006). Platelet-rich fibrin (PRF): A second-generation platelet concentrate. Part III: Leucocyte activation: A new feature for platelet concentrates?. Oral. Surgery, Oral Medicine, Oral Pathology, Oral Radiology Endod..

[19] Dohan DM (2006). Platelet-rich fibrin (PRF): A second-generation platelet concentrate. Part II: Platelet-related biologic features. *Oral Surgery*, *Oral Medicine*, *Oral Pathology*. Oral. Radiology Endod..

[20] Preeja C, Arun S (2014). Platelet-rich fibrin: Its role in periodontal regeneration. Saudi J. Dental Res..

[21] Bodhare Girish H., Kolte Abhay P., Kolte Rajashri A., Shirke Prerna Y. (2018). Clinical and radiographic evaluation and comparison of bioactive bone alloplast morsels when used alone and in combination with platelet‐rich fibrin in the treatment of periodontal intrabony defects—A randomized controlled trial. Journal of Periodontology.

[22] Zhou S (2018). Efficacy of Adjunctive Bioactive Materials in the Treatment of Periodontal Intrabony Defects: A Systematic Review and Meta-Analysis. Biomed. Res. Int..

[23] Pan J (2019). Effect of platelet-rich fibrin on alveolar ridge preservation: A systematic review. J. Am. Dent. Assoc..

[24] Li A (2019). Additive effectiveness of autologous platelet-rich fibrin in the treatment of intrabony defects: A PRISMA-compliant meta-analysis. Med..

[25] Sharma A, Pradeep AR (2011). Treatment of 3-wall intrabony defects in patients with chronic periodontitis with autologous platelet-rich fibrin: a randomized controlled clinical trial. J. Periodontol..

[26] Ajwani H (2015). Comparative evaluation of platelet-rich fibrin biomaterial and open flap debridement in the treatment of two and three wall intrabony defects. J. Int. Oral. Health.

[27] Joseph DB, Borer JG, De Filippo RE, Hodges SJ, McLorie GA (2014). Autologous cell seeded biodegradable scaffold for augmentation cystoplasty: phase II study in children and adolescents with spina bifida. J. Urol..

[28] Chandradas ND, Ravindra S, Rangaraju VM, Jain S, Dasappa S (2016). Efficacy of platelet rich fibrin in the treatment of human intrabony defects with or without bone graft: A randomized controlled trial. J. Int. Soc. Prev. Community Dent..

[29] Sezgin Y, Uraz A, Taner IL, Culhaoglu R (2017). Effects of platelet-rich fibrin on healing of intra-bony defects treated with anorganic bovine bone mineral. Braz. Oral. Res..

[30] Jadhav GR, Shah D, Raghvendra SS (2015). Autologus Platelet Rich Fibrin aided Revascularization of an immature, non-vital permanent tooth with apical periodontitis: A case report. J. Nat. Sci. Biol. Med..

[31] Dohan DM (2006). Platelet-rich fibrin (PRF): a second-generation platelet concentrate. Part II: platelet-related biologic features. Oral. Surg. Oral Med. Oral Pathol. Oral Radiol. Endod..

[32] Chang YC, Zhao JH (2011). Effects of platelet-rich fibrin on human periodontal ligament fibroblasts and application for periodontal infrabony defects. Aust. Dent. J..

[33] Duan X (2018). Study of platelet-rich fibrin combined with rat periodontal ligament stem cells in periodontal tissue regeneration. J. Cell Mol. Med..

[34] Galav S (2016). Comparative evaluation of platelet-rich fibrin and autogenous bone graft for the treatment of infrabony defects in chronic periodontitis: Clinical, radiological, and surgical reentry. Indian. J. Dent. Res..

[35] Cicciù, M., Herford, A. S., Juodžbalys, G. & Stoffella, E. Recombinant Human Bone Morphogenetic Protein Type 2 Application for a Possible Treatment of Bisphosphonates-Related Osteonecrosis of the Jaw. *Journal of Craniofacial Surgery* **23** (2012). 10.1097/SCS.0b013e31824dbdd4 22565901

[36] Herford, A. S. & Cicciù, M. Recombinant Human Bone Morphogenetic Protein Type 2 Jaw Reconstruction in Patients Affected by Giant Cell Tumor. *Journal of Craniofacial Surgery* **21** (2010). 10.1097/SCS.0b013e3181f502fa 21119472

[37] Scott Herford A (2016). rhBMP-2 applied as support of distraction osteogenesis: A split-mouth histological study over nonhuman primates mandibles. Int. J. Clin. Exp. Med..

[38] Poli PP, Beretta M, Cicciù M, Maiorana C (2014). Alveolar ridge augmentation with titanium mesh. A retrospective clinical study. Open. Dent. J..

[39] Hatakeyama I, Marukawa E, Takahashi Y, Omura K (2014). Effects of platelet-poor plasma, platelet-rich plasma, and platelet-rich fibrin on healing of extraction sockets with buccal dehiscence in dogs. Tissue Eng. Part. A.

[40] Silva RF, Carmona JU, Rezende CM (2012). Comparison of the effect of calcium gluconate and batroxobin on the release of transforming growth factor beta 1 in canine platelet concentrates. BMC Vet. Res..

[41] Masuki H (2016). Growth factor and pro-inflammatory cytokine contents in platelet-rich plasma (PRP), plasma rich in growth factors (PRGF), advanced platelet-rich fibrin (A-PRF), and concentrated growth factors (CGF). Int. J. Implant. Dent..

[42] Pakyari M, Farrokhi A, Maharlooei MK, Ghahary A (2013). Critical Role of Transforming Growth Factor Beta in Different Phases of Wound Healing. Adv. Wound Care.

[43] Pierce GF, Mustoe TA, Altrock BW, Deuel TF, Thomason A (1991). Role of platelet-derived growth factor in wound healing. J. Cell Biochem..

[44] Johnson KE, Wilgus TA (2014). Vascular Endothelial Growth Factor and Angiogenesis in the Regulation of Cutaneous Wound Repair. Adv. Wound Care.

[45] Yoon JS, Lee SH, Yoon HJ (2014). The influence of platelet-rich fibrin on angiogenesis in guided bone regeneration using xenogenic bone substitutes: a study of rabbit cranial defects. J. Craniomaxillofac Surg..

[46] Smith PC, Cáceres M, Martínez C, Oyarzún A, Martínez J (2015). Gingival Wound Healing:An Essential Response Disturbed by Aging?. J. Dental Res..

[47] Gonzalez ACdO, Costa TF, Andrade ZdA, Medrado ARAP (2016). Wound healing - A literature review. An. Brasileiros de. Dermatologia.

[48] Wang Z-S (2016). The use of platelet-rich fibrin combined with periodontal ligament and jaw bone mesenchymal stem cell sheets for periodontal tissue engineering. Sci. Rep..

[49] Cekici A, Kantarci A, Hasturk H, Van Dyke TE (2014). Inflammatory and immune pathways in the pathogenesis of periodontal disease. Periodontology 2000.

[50] Kanno E (2011). Wound healing in skin promoted by inoculation with Pseudomonas aeruginosa PAO1: The critical role of tumor necrosis factor-alpha secreted from infiltrating neutrophils. Wound Repair. Regen..

[51] Grigoriadou ME, Koutayas SO, Madianos PN, Strub JR (2010). Interleukin-1 as a genetic marker for periodontitis: review of the literature. Quintessence Int..

[52] Dohan Ehrenfest DM, Del Corso M, Diss A, Mouhyi J, Charrier J-B (2010). Three-Dimensional Architecture and Cell Composition of a Choukroun’s Platelet-Rich Fibrin Clot and Membrane. J. Periodontology.

[53] Ghanaati S (2014). Advanced Platelet-Rich Fibrin: A New Concept for Cell-Based Tissue Engineering by Means of Inflammatory Cells. J. Oral. Implantology.

[54] Keswani D, Pandey RK (2013). Revascularization of an immature tooth with a necrotic pulp using platelet-rich fibrin: a case report. Int. Endod. J..

[55] Suzuki S, Morimoto N, Ikada Y (2013). Gelatin gel as a carrier of platelet-derived growth factors. J. Biomater. Appl..

[56] Ji B (2014). The Combination Use of Platelet-Rich Fibrin and Treated Dentin Matrix for Tooth Root Regeneration by Cell Homing. Tissue Eng. Part. A.

[57] Fujioka-Kobayashi M (2017). Optimized Platelet-Rich Fibrin With the Low-Speed Concept: Growth Factor Release, Biocompatibility, and Cellular Response. J. Periodontology.

[58] Miron, R. J. *et al*. Comparison of platelet-rich fibrin (PRF) produced using 3 commercially available centrifuges at both high (~ 700 g) and low (~ 200 g) relative centrifugation forces. *Clinical Oral Investigations*, 10.1007/s00784-019-02981-2 (2019). 10.1007/s00784-019-02981-2 31321574

[59] El Bagdadi K (2019). Reduction of relative centrifugal forces increases growth factor release within solid platelet-rich-fibrin (PRF)-based matrices: a proof of concept of LSCC (low speed centrifugation concept). Eur. J. Trauma. Emerg. Surg..

[60] Choukroun J, Ghanaati S (2018). Reduction of relative centrifugation force within injectable platelet-rich-fibrin (PRF) concentrates advances patients’ own inflammatory cells, platelets and growth factors: the first introduction to the low speed centrifugation concept. Eur. J. Trauma. Emerg. Surg..

[61] Miron R, Choukroun J, Ghanaati S (2018). Controversies related to scientific report describing g-forces from studies on platelet-rich fibrin: Necessity for standardization of relative centrifugal force values. Int. J. Growth Factors Stem Cell Dent..

[62] Miron RJ, Pinto NR, Quirynen M, Ghanaati S (2019). Standardization of relative centrifugal forces in studies related to platelet-rich fibrin. J. Periodontology.

[63] Kobayashi E (2016). Comparative release of growth factors from PRP, PRF, and advanced-PRF. Clin. Oral. Investigations.

[64] Ghanaati S (2018). Application of liquid platelet-rich fibrin for treating hyaluronic acid-related complications: A case report with 2 years of follow-up. Int. J. Growth Factors Stem Cell Dent..

[65] Jianpeampoolpol B, Phuminart S, Subbalekha K (2016). Platelet-rich Fibrin Formation was delayed in Plastic Tubes. Br. J. Med. Med. Res..

[66] Bracken MB (2009). Why animal studies are often poor predictors of human reactions to exposure. J. R. Soc. Med..

[67] Di Bello A (2014). Periodontal disease associated with red complex bacteria in dogs. J. Small Anim. Pract..

[68] Sturgeon A, Pinder S, Costa M, Weese J (2014). Characterization of the oral microbiota of healthy cats using next-generation sequencing. Veterinary J..

[69] Dewhirst FE (2012). The canine oral microbiome. PLoS One.

[70] Heitz-Mayfield LJA, Trombelli L, Heitz F, Needleman I, Moles D (2002). A systematic review of the effect of surgical debridement vs. non-surgical debridement for the treatment of chronic periodontitis. J. Clin. Periodontology.

[71] Watanabe T (2017). An Evaluation of the Accuracy of the Subtraction Method Used for Determining Platelet Counts in Advanced Platelet-Rich Fibrin and Concentrated Growth Factor Preparations. *Dentistry*. J..

[72] Aroca S, Keglevich T, Barbieri B, Gera I, Etienne D (2009). Clinical evaluation of a modified coronally advanced flap alone or in combination with a platelet-rich fibrin membrane for the treatment of adjacent multiple gingival recessions: a 6-month study. J. Periodontol..

[73] Löe H (1967). The Gingival Index, the Plaque Index and the Retention Index Systems. J. Periodontology.

[74] Laster L, Laudenbach KW, Stoller NH (1975). An Evaluation of Clinical Tooth Mobility Measurements. J. Periodontology.

[75] Persson RE (2003). Comparison between panoramic and intra-oral radiographs for the assessment of alveolar bone levels in a periodontal maintenance population. J. Clin. Periodontol..

[76] Balci Yuce H, Toker H, Goze F (2014). The histopathological and morphometric investigation of the effects of systemically administered boric acid on alveolar bone loss in ligature-induced periodontitis in diabetic rats. Acta Odontol. Scand..

[77] Takahashi K (1994). Assessment of interleukin-6 in the pathogenesis of periodontal disease. J. Periodontol..

[78] Furtado ARR, Constantino-Casas F (2013). Histopathology inflammation scoring and classification in 34 dogs with inflammatory nasal disease. Veterinary Rec..

[79] Natarajan S, Remick DG (2008). The ELISA Standard Save: Calculation of sample concentrations in assays with a failed standard curve. J. Immunological Methods.

